# Optimizing Acellular Dermal Matrix Integration in Heterologous Breast Reconstructive Surgery: Surgical Tips and Post-Operative Management

**DOI:** 10.3390/medicina59071231

**Published:** 2023-06-30

**Authors:** Glenda Caputo, Anna Scarabosio, Jacopo Di Filippo, Filippo Contessi Negrini, Roberta Albanese, Sebastiano Mura, Pier Camillo Parodi

**Affiliations:** 1 Clinic of Plastic and Reconstructive Surgery, Academic Hospital of Udine, Department of Medical Area (DAME), University of Udine (Italy), 33100 Udine, Italy; sblenda@yahoo.it (G.C.); jacopo.difilippo38@gmail.com (J.D.F.); f.contessi@icloud.com (F.C.N.); piercamillo.parodi@uniud.it (P.C.P.); 2 Clinic of Plastic and Reconstructive Surgery, Ospedale Santa Maria della Misericordia, 33100 Udine, Italy; roberta.albanese@asufc.sanita.fvg.it (R.A.); banomu88@live.it (S.M.)

**Keywords:** prepectoral reconstruction ADM, dermal matrix, ADM integration, breast reconstruction, implant-based reconstruction, heterologous reconstruction, breast surgery

## Abstract

*Background and Objective*: Prepectoral implant placement in breast reconstruction is currently a must-have in the portfolios of breast surgeons. The introduction of new tools and conservative mastectomies is a game changer in this field. The prepectoral plane usually goes hand-in-hand with the ADM wrapping of the implant. It is a cell-free dermal matrix comprising a structurally integrated basement membrane complex and an extracellular matrix. The literature reports that ADMs may be useful, but proper patient selection, surgical placement, and post-operative management are essential to unlock the potential of this tool, as these factors contribute to the proper integration of the matrix with surrounding tissues. *Materials and Methods*: A total of 245 prepectoral breast reconstructions with prostheses or expanders and ADMs were performed in our institution between 2016 and 2022. A retrospective study was carried out to record patient characteristics, risk factors, surgical procedures, reconstructive processes, and complications. Based on our experience, we developed a meticulous reconstruction protocol in order to optimize surgical practice and lower complication rates. The DTI and two-stage reconstruction were compared. *Results*: Seroma formation was the most frequent early complication (less than 90 days after surgery) that we observed; however, the majority were drained in outpatient settings and healed rapidly. Secondary healing of wounds, which required a few more weeks of dressing, represented the second most frequent early complication (10.61%). Rippling was the most common late complication, particularly in DTI patients. After comparing the DTI and two-stage reconstruction, no statistically significant increase in complications was found. *Conclusions*: The weakness of prepectoral breast reconstruction is poor matrix integration, which leads to seroma and other complications. ADM acts like a graft; it requires firm and healthy tissues to set in. In order to do so, there are three key steps to follow: (1) adequate patient selection; (2) preservative and gentle handling of intra-operative technique; and (3) meticulous post-operative management.

## 1. Introduction

Prepectoral implant placement in breast reconstruction is currently a must-have in the portfolios of breast surgeons. This procedure is not really brand new; Snyderman et al. described it in 1971 [1]. After the first attempt, it was noted that complications such as capsular contracture, implant exposure, and malposition were commonly observed due to the subcutaneous placement of the implant [2,3]. The simultaneous evolution of mastectomy procedures, with muscle-sparing technique, provided the chance to include an extra layer of tissue to cover the implant: pectoralis major and serratus anterior muscles. The first description of submuscular breast reconstruction was in 1981, and its advantages were immediately appreciated; implant exposure also decreased immediately. Nevertheless, some issues remained, such as breast animation deformity, capsular contracture, and poor aesthetic outcomes [2,4,5,6]. Dual-plane placement is an attempt to achieve a more natural reconstructed breast and a single-stage reconstruction. To sustain the mammary lower pole, an acellular dermal matrix (ADM) was introduced. Currently, this approach is adequate in a few cases [7,8].

In this context, modern prepectoral breast reconstruction was developed. It significantly differs from Snyderman’s technique, and the introduction of ADMs and conservative mastectomies is considered a game changer in this field [9,10,11,12]. As a matter of fact, the recent success of this procedure is rooted in tools and techniques that have been developed over the last 20 years [3].

First, oncoplastic breast surgery has drastically evolved. Starting from the highly debulking Halstead’s mastectomy to Madden and Patey’s variations, the surgery now involves skin-sparing, nipple-sparing, and skin-reducing procedures. When these techniques are followed adequately, they leave a proper and well-vascularized mastectomy flap, while simultaneously maintaining oncological safety. A well-performed mastectomy represents the first key point in a successful prepectoral reconstruction, which may be assessed [2,13].

The prepectoral plane usually goes together with the ADM wrapping of the implant. ADM is a cell-free dermal matrix comprising a structurally integrated basement membrane complex and an extracellular matrix, in which collagen bundles and elastic fibers are the main components. Acellular dermal matrices (ADMs) have been developed from a variety of sources, including human (HADM), porcine (PADM), and bovine (BADM), using multiple processing protocols [14,15,16]. In Table 1 the most common are shown.

This tool permits fixation of the implant in the pocket and reduces implant exposure risk [12,17]. From a long-term perspective, it seems to lower capsular contracture, and no animation deformity has been reported due to the prepectoral placement [11,18]. On the other hand, some patients may need lipofilling revision surgery to improve breast contour or rippling [19].

The literature reports that ADMs may be useful, but proper patient selection, surgical placement, and post-operative management are essential to fully benefit from the potential of this tool. All of these factors facilitate the proper integration of the matrix with the surrounding tissues [20,21].

## 2. Materials and Methods

Two hundred and forty-five prepectoral breast reconstructions with prostheses or expanders and ADMs were performed at our institution between 2016 and 2022. A retrospective study was carried out to record patient characteristics, risk factors, surgery procedures, reconstructive processes, and complications. 

Based on our experience, we developed a meticulous reconstruction protocol in order to optimize surgical practice and lower complication rates. All of these were recorded and classified as soon as they occurred in the first 90 days post-operation, or later. Moreover, seroma was defined as minor when treated in outpatient settings or major when a second surgery was needed.

In order to evaluate patients who underwent immediate breast reconstruction and those who required two-stage reconstructions with tissue expander as a single group, a Chi-squared test was performed to compare complication rates observed.

1. Patient selection

Several relative contraindications are reported in the literature, such as systemic comorbidities (uncontrolled diabetes mellitus and connective tissue diseases), active or past smoking, high/low BMI (body mass index), and previous radiotherapy [20,22,23]. In fact, any risk factor that may negatively influence the vascular supply to the mastectomy flaps should be a relative contraindication. 

The pinch test is always performed. This easy evaluation helps to approximately predict the mastectomy flap thickness. In addition, magnetic resonance imaging (MRI) may also help in this assessment; usually, the lowest thickness is found around the NAC. Moreover, if the patients have very ptotic or large breasts, they may not be suitable candidates. 

All these aspects are evaluated during the pre-operative consultation, defining the patient’s eligibility for prepectoral implant positioning and matrix placement. V-Breast (a method to quantify breast volume based on mammary gland measurements) is calculated, and the implant size range is chosen [24].

2. Surgical procedure

The following pre-operative markings are drawn: the inframammary fold (IMF), midline, midclavicular line, jugular groove, and the contour of the original breast. Antibiotic prophylaxis is always administered 30 min pre-operatively (one endovenous shot of cefazolin 2 g). Then, skin-sparing or nipple-sparing mastectomies and lymph node biopsy or dissection were fully performed by expert breast surgeons. The surgical plane of the mastectomy is between the mammary gland and the subcutaneous tissue, where Cooper’s ligaments lie, in order to preserve the subdermal plexus. Lymph node biopsy and retro-areolar breast tissue are immediately sent for a histopathological examination. If they are both free from disease, the reconstruction time begins. On the other hand, if the axilla lymph node is positive, dissection is performed, and if the exam reports cancer residues in the retro-areolar tissue, the nipple-areola complex (NAC) is removed. In the case of lymph node positivity, it is preferable to perform a two-stage reconstruction due to the high probability of adjuvant radiotherapy [25].

The first step in a good reconstructive procedure is the full examination of the mastectomy flaps. In our institution, a clinical evaluation of the flap viability (color, time of refilling, and thickness) is commonly performed before the reconstruction. Whenever the flap appears viable, an implant is placed in the prepectoral plane. In some cases, an expander may be used to reduce pressure on the delicate vascular dermal plexus. The hemostasis is revised, the neo-pocket is accurately checked, and implant sizers are used to choose the proper implant. 

Then, the ADM-Braxon is unpacked and prepared on the back table in a fully sterile area. Therefore, rehydration is required. This process is performed using ambient-temperature sterile saline solution. It takes from 1 to 30 min depending on the ADM type (Table 1). When the ADM acquires a soft and pliable aspect, the implant is accurately wrapped. In the case of Braxon fast use, the front and back sheets are closed with a double continuous resorbable suture (Vicryl 2/0). The exceeding matrix is trimmed to create a perfectly adherent shell. Small holes are made with a narrow scalpel, allowing the drainage of serum between the ADM and the fat tissue of the mastectomy flaps. It is also possible to place, where necessary, some fixing sutures attaching the ADM to the mastectomy subcutaneous tissue while being careful not to damage the implant with the needle. Usually, four resorbable sutures (Vicryl 2/0) are prepared and fixed to the ADM (leaving the suture thread long and with the needle attached) at 2, 4, 8, and 10 o’clock. The mammary pocket is then washed with a triple Betadine solution [26]. Thus, the ADM implant coated and loaded with these sutures is placed in the pocket, and the patient is placed in a sitting position with the arm adducted. Four or more anchoring sutures are fixed in accordance with the grade of ptosis, and other quilting sutures between the ADM and mastectomy flaps are secured through the holes previously made on the ADM on demand. Particular attention is paid to the fixation of the nipple–areola complex in the central position of the implant by properly tightening the sutures. 

When PRS meshed or Exashape is used, the procedure is quite similar. They are both meshed, and while PRS meshed has flexible and on-demand wrapping and sutures, Exashape has its own assembly system. These placements are fully described in Videos S1 (Braxon), S2 (PRS meshed), and S3 (Exashape). 

Two subcutaneous suction drains are always inserted: one draining the upper pole and the other draining the lower pole. Once properly placed, the skin suture is completed with two or three suture layers.

3. Post-operative management

Correct and meticulous post-operative management of this reconstruction is crucial. Immediately after the surgical procedure, an anterior closure compressive bra associated with an upper pole belt is placed on the breasts, reducing dead spaces and enhancing the cohesion between the mastectomy flaps, dermal matrix, and implant. Reduced upper-limb movement for at least the first 2–3 weeks is strongly suggested. In our practice, the arm is usually not fully blocked, but patients are carefully informed and trained by the hospital staff on how to move; going to the gym, weightlifting, jumping, and car driving are fully forbidden for at least a month. Daily activities, personal care, and light stretching of the arms are allowed. An information sheet is also supplied to stress the importance of limiting movements. The hospital stay lasts 2–3 days, depending on the general patient status and drainage quantity. Then, the post-operative period can be managed on an outpatient basis. Usually, medications are administered two times a week. The main concern is regarding drainage management. They are maintained for at least 7 days, more commonly between 10 and 18 days when there is a relative certainty that wounds are properly closed and the dermal matrix fully began its integration. Standard thromboembolic prophylaxis (enoxaparin 4000 U.I. daily) is administered for three weeks post-operatively. A compressive bra must be worn 24/7 for at least a month and then gradually reduced.

**Figure 1:**
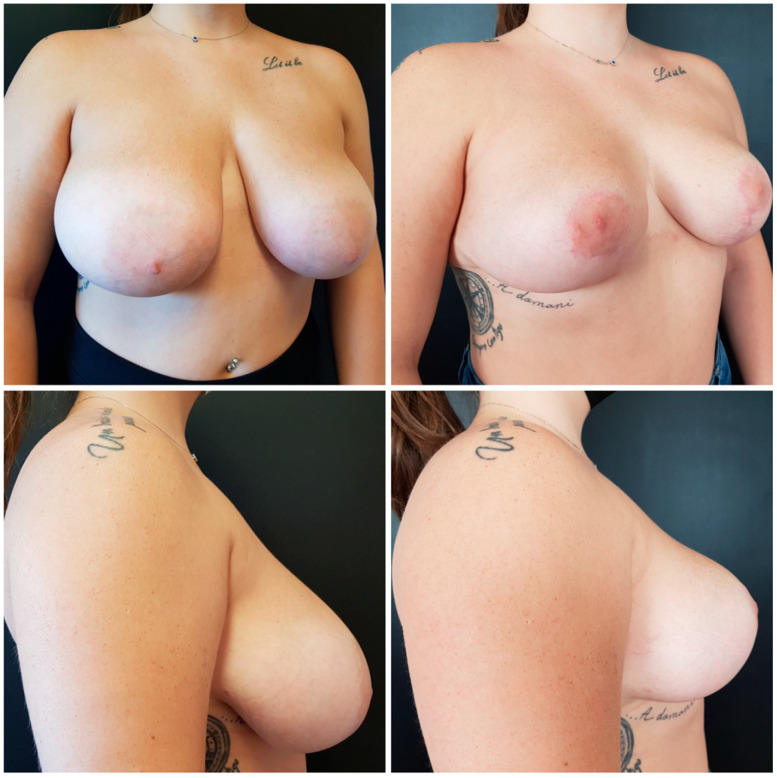
Before and after pictures of a young patient who underwent skin-reducing mastectomy and DTI reconstruction.

**Figure 2:**
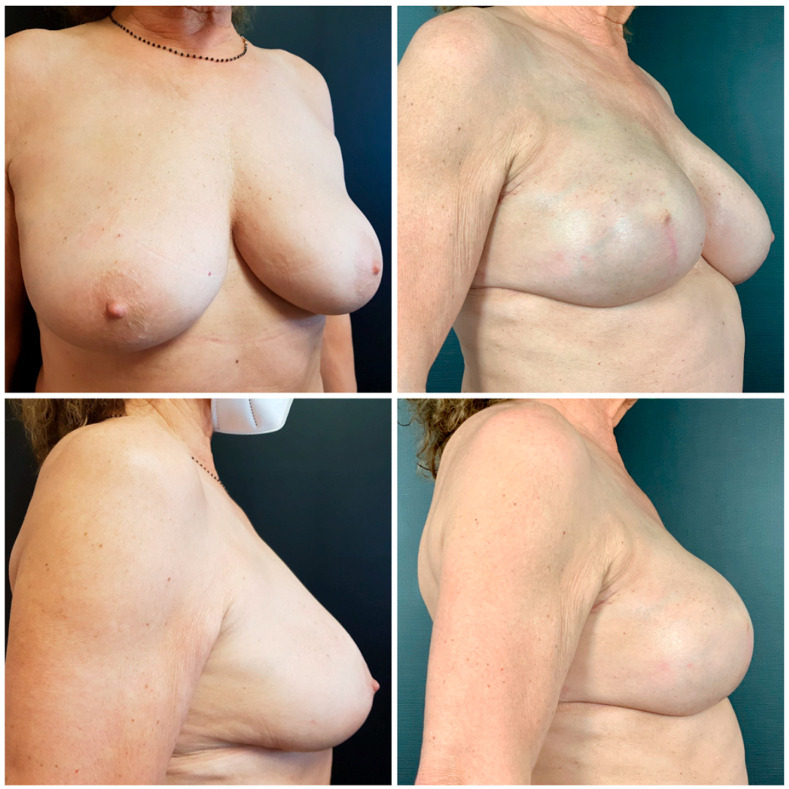
Before and after pictures of an older patient who underwent skin-reducing mastectomy with two-stage reconstruction.

Pre-operative and post-operative patient pictures are meticulously taken and compared (Figure 1 and Figure 2).

## 3. Results

In our institution, 245 prepectoral breast reconstructive procedures were performed between 2016 and 2022. Table 2 fully presents the main characteristics.

Among these procedures, 195 (79.6%) were DTI reconstructions and 50 (20.4%) were two-stage reconstructions. Only twenty-one 21 (8.57%) were prophylactics. All the patients were females, with a median age of 50 years and an average BMI of 23.1.

Some risk factors were present in the selected population: 13.5% of patients were smokers, and 2.4% had diabetes. One hundred and seventy-six patients underwent nipple-sparing mastectomy, 57 underwent skin-sparing, and 12 underwent skin-reducing procedures. Lymph node surgery was performed on 182 patients, most of whom underwent only sentinel node biopsy, whereas 42 patients had full axillary dissection. Twenty-one patients did not undergo lymph node surgery on the mastectomy side because they were prophylactic. 

Fifty-five patients underwent radiotherapy: 28 pre-operatively and 27 post-operatively. In the second group, 14 patients underwent DTI, and 13 underwent a two-stage reconstruction. Chemotherapy was necessary for 88 patients. Our follow-up period was quite long (24 months on average), and adherence was quite high (9.3% of lost patients).

Early (less than 90 days from surgery) and late complications (more than 90 days from surgery) are fully described in Table 3. Although seroma formation was the most frequent early complication, most of the minor seromas were drained in outpatient settings and healed rapidly. Wound dehiscence was treated similarly, which needed a few more weeks of dressing. 

In Table 3, complications are shown for direct-to-implant reconstructions and two-stage reconstructions. The latter is usually performed when, during intra-operative assessment, the mastectomy flap quality is deemed suboptimal, or when large implants are required. This choice lowers the skin tension and thus reduces the risk of wound dehiscence and flap necrosis, which would otherwise flourish in these patients. Lower-quality mastectomy flaps also result in a higher risk of reconstruction failure. However, none of the above-mentioned complications showed a statistically significant difference between the two groups, allowing us to consider a single group of patients.

## 4. Discussion

The use of implant-based reconstruction has grown rapidly over the last 20 years. Compared to microsurgical options, this procedure seems to be faster, easier, and equally safe. The first heterologous reconstruction dates back to 1963, when the first silicone prosthesis was placed as a delayed procedure following mastectomy [1]. Then, it was temporarily abandoned due to its complication rates and poor aesthetic outcomes. In the past, prosthesis was usually placed in a submuscular plane in order to reduce exposure and contracture rates. On the other hand, this technique resulted in a higher bleeding risk, post-operative pain, and limitation in shoulder movements [3,18]. 

In this context, prepectoral reconstruction regained a new life, and it represents the first option in many breast units for selected patients [20,26,27]. The main limitation is the cost of ADMs, but the advantages are clear. In this procedure, breast implants are placed in a pocket formed by the mastectomy flaps and the chest wall muscles, which are no longer elevated. Consequently, the main issue is the evaluation of the viability of the residual breast envelope. It must be adequately perfused in order to avoid major complications, such as skin necrosis and implant exposure. Patient selection and intra-operative flap evaluation are the cornerstones in this set.

From this perspective, matrices were first introduced assuming that these could improve, sustain, and thicken the mastectomy flaps. The results arrived quite soon: the implant exposure rate decreased and a better aesthetic result was achieved. However, as any improvement may have a downside, matrices have been demonstrated to be associated with a higher risk of seroma formation, implant loss, and revision surgeries [10,14]. Currently, this technique has improved, with an acceptable complication rate [12,28]. Moreover, ADM placement seems to reduce capsular contracture and protect against postmastectomy radiation therapy issues [11,17]. 

In our four-year case series, we did not observe any significant difference in complication rates between the DTI and two-stage reconstruction groups, even though some results were very close to the significance (hematoma, flap necrosis, wound dehiscence, and implant loss). A total of 10 out of 245 patients developed a seroma that required revision surgery. Minor seromas remained quite frequent (16.7%) but were managed on an outpatient basis, usually drained with ultrasound guidance only once or twice a month after the major surgery. This is found to be most common when implants are larger than 400 cc (*p* < 0.005). Hematoma (2–3%) and infections (5–9%) are in line with other DTI procedures described in the literature [5,9,21]. In late complications, the most relevant is rippling, which is present in one-third of reconstructed breasts. This side effect may seem costly but represents the price to pay for the prepectoral plane, which leads to a more natural breast and better function of the upper limbs over the years. Moreover, most of the time, this may be solved with one or two lipografts surgeries [29]. 

Understanding and optimizing the integration process of breast matrices can drastically reduce the main complications, particularly seromas. To promote integration, some characteristics of ADMs have to be taken into consideration. ADMs are water-insoluble matrices that originate from the decellularization process of human, bovine, or porcine connective tissues. Once implanted, they function as scaffolds for donor-site cells to facilitate subsequent incorporation and revascularization.

Some in vivo studies on mice have shown that these membranes can induce a biological activation in the surrounding tissues. Specifically, the induction of adipogenic stimulation is morphological-dependent on the porosity, thickness, and swelling ratio of the membrane. The deposition of newly formed adipose tissue may play a key role in forming a well-organized tissue architecture around the implant [30]. 

The matrix can be considered a soft connective tissue graft. The main step to success is adequate adhesion between the mastectomy flaps and the matrix. For example, patients with large and ptotic breasts may not be ideal candidates. In these patients, compromised viability of such wide flaps is quite common. In addition, excessive movement of the matrix in the pocket makes the interface between the mastectomy flap and the ADM unstable, limiting the integration process. This problem may be reduced by the skin-reducing technique [31,32], which reduces the extension of the mastectomy flaps to perfectly fit the implant in the pocket.

When adequate tissue contact is present, the activation of the surroundings may initiate the incorporation process. This is probably followed by imbibition, recellularization, and revascularization of the ADM.

Below, we describe the essential elements for the proper management of these patients.

First, it is recommended to choose patients who may have adequate microcirculation; patients who are active smokers and have uncontrolled diabetes, connective tissue diseases, and obesity should be avoided. Second, an accurate intra-operative evaluation must be performed. Flap vascularization is mainly based on the dermal plexus, which mainly receives blood from the internal mammary perforators and the fifth anterior intercostal artery perforator (AICAP). These, if preserved, may prevent NAC necrosis [33].

There should be active bleeding from the mastectomy flap margins, adequate refill, temperature, and skin color. The dermis layer should not be visible on the mastectomy flaps. In any doubtful case, indocyanine green may be used. This technique is surely very specific, but its high cost and poor availability limit its widespread use [34,35,36]. 

As with skin grafts, the delicate integration of ADMs must be facilitated all the way. This is the reason why developing a protocol to follow is required.

Another main issue is increasing the contact surface between a graft and the host tissue, avoiding sliding movements between them. This is why it is advised to continue drainage for at least a week, limit arms movements for two weeks, and wear a compressive bra for a month. Moreover, in order to reduce dead spaces, implants should be volumetrically adequate for the pocket. If the original breast is ptotic or large, reducing the skin envelope (skin-reducing mastectomy) allows for better surface adherence. Another useful tip involves placing a compressive bra with an upper pole belt immediately after surgery [37,38]. 

The last element to improve the integration process consists of developing the perfect matrix. This should be immuno-inert and quickly vascularized. Unfortunately, the latter two characteristics are unshakable: neovascularization is dependent on a coordinated immune response. Thus, the immune response must be present but tempered, perfectly targeted, and ordered. This would enhance rapid vascularization, limiting complications. 

A non-integrated matrix is the main cause of seroma formation and chronic inflammation. This inflammatory response becomes particularly clear in the case of red breast syndrome (RBS), which is a recently identified clinical entity characterized by non-infectious erythema associated with the use of an acellular dermal matrix (ADM) after post-mastectomy reconstruction. 

Once an ADM is properly integrated, the road is downhill. The literature reports a decrease in capsular contracture, which seems to be based on the creation of an interface between the implant and surrounding tissue, which “camouflages” the implant, mitigating the foreign body reaction [27]. This theory may explain the decrease in inflammatory cells and myofibroblasts around the implant. Better aesthetic outcomes in terms of capsular contracture have already been demonstrated [11,39].

## 5. Conclusions

Prepectoral breast reconstruction using ADM is currently quite common. Its weakness, which is often the cause of poor results and complications, is poor matrix integration. There are still a few main issues to solve in terms of ADM usage. First, the preparation costs are high, which limits their use. Moreover, seroma formations are more frequent compared to non-ADM reconstruction. Further studies are needed to fully understand the matrix integration processes and optimize their indications.

ADM acts like a graft; it requires firm and healthy tissues to set in. In order to do so, there are three key steps to follow: (1) adequate patient selection; (2) preservative and gentle handling of intra-operative technique; and (3) meticulous post-operative management. These should increase proper adhesion between the tissue and matrix, thereby implementing the integration process and lowering complication rates.

## Figures and Tables

**Table 1:** Biological matrices used in breast reconstruction.

Name	Source	Manufacturer	Country	Hydration Time	Decellularization	Sterilization
AlloDerm	Human	Lifecell	USA	30 min	Not available	Not available
FlexHD	Human	Ethicon, Inc.	USA	Ready to use	Hypertonic bath	Ethanol and peracetic acid
DermACELL	Human	LifeNet Health	USA	Ready to use	Matracell	γ-irradiation
Strattice	Porcine	Lifecell	USA	2–3 min	Sodium chloride and detergent	Electron beam gamma radiation
Surgimend PRS	Fetal bovine	TEI Biosciences	USA	3–4 min	Not available	
Braxon	Porcine	DECOmed	Italy	5–6 min	Not available	Not available
Exashape	Bovine pericardium	Advanced Bioconcept	Italy	5–6 min	Hypertonic solution	γ-irradiation

**Table 2:** Population and surgical procedure data.

Age at surgery, years, median (range)	50 (39–65)
Follow-up, months, median (range)	24 (24–63)
Lost patients	23 (9.4%)
BMI, kg/m^2^, median (range)	23.1 (17–35.8)
Overweight (BMI > 25)	48 (19.6)
Active smoking, n (%)	33 (13.5)
Comorbidity, n (%)	
Hypertension	21 (8.6)
Diabetes	6 (2.4)
Others	0
Removed breast tissue, g	
Median (range)	308.4 (83–450)
Missing	18
Mastectomy procedure	
Nipple-sparing	176
Skin-sparing	57
Skin-reducing	12
Reconstructive time	
DTI	195 (79.1)
Two-stage	50 (20.9)
Implant size, cc median (range) *	348.7 (120–650)
ADM, n (%)	
Braxon	196 (80.00)
Surgimen PRS meshed	44 (17.96)
Exashape	5 (2.04)
Breast reconstruction side, n (%)	
Right	128 (52.2)
Left	117 (47.7)
Chemotherapy, n (%)	88 (35.9)
Neoadjuvant	23
Adjuvant	65
Radiotherapy, n (%)	
Pre-operative	28 (11.4)
Post-operative	27 (11.02)
Axillary lymph node surgery, n (%)	
Sentinel node biopsy	182 (74.29)
Dissection	42 (17.14)
None	21 (8.57%)
Drain duration, days, median (range)	12.96 (9–24)

Legend: BMI—body mass index; DTI—direct to implant. * indicates that in the expander group, the final expansion volume is recorded.

**Table 3:** Complications in DTI and two-stage procedures.

Early Complications	DTI—Mean	Two-Stage—Mean	Total (%)	*p*-Value *
Seroma				
Minor	33	8	41 (16.73)	0.88
Major	9	1	10 (4.08)	0.40
Hematoma	21	0	21 (8.57)	0.02
Infection	7	0	7 (2.85)	0.17
Flap necrosis	22	1	23 (9.38)	0.04
Wound dehiscence	25	1	26 (10.61)	0.03
**Late Complications**				
Animation	1	1	2 (0.81)	0.30
Rippling	69	15	84 (34.28)	0.47
Rotation	11	1	12 (4.90)	0.29
Failure (implant loss)	9	7	16 (6.53)	0.02

* *p*-value was calculated using a Chi-squared test and by comparing DTI and two-stage.

## Data Availability

Data are conserved in hospital system.

## Supplementary Materials

The following supporting information can be downloaded at: https://www.mdpi.com/article/10.3390/medicina59071231/s1, Video S1: Braxon preparation; Video S2: PRS meshed preparation; Video S3: Exashape preparation.
